# Help-seeking behavior among miscarrying women with and without post-miscarriage health problems in Ghana

**DOI:** 10.1371/journal.pgph.0002458

**Published:** 2023-10-09

**Authors:** Samuel Kwaku Essien, Batholomew Chireh, Peter Kwabena Essien

**Affiliations:** 1 Department of Health Sciences, Lakehead University, Thunder Bay, ON, Canada; 2 EPID@Work Research Institute, Lakehead University, Thunder Bay, ON, Canada; 3 Department of Psychology, Lakehead University, Thunder Bay, ON, Canada; 4 Kwame Nkrumah University of Science and Technology, Kumasi, Ghana; Dalhousie University, CANADA

## Abstract

Understanding how frequently women seek assistance after experiencing a miscarriage could potentially help address unmet needs in managing post-miscarriage health problems (PMHP). However, most studies focus primarily on the causes and effects of PMHP and neglect the influence of help-seeking behavior on PMHP. This study examined help-seeking behavior among women who have experienced a miscarriage in Ghana, whether they sought help from healthcare professionals or not, and the impact it had on post-miscarriage health problems (PMHP). The study analyzed subsample data (N = 1,843) from the 2017 Ghana maternal health survey of miscarrying women aged 15–49 years who answered questions on help-seeking after a miscarriage from 900 clusters in ten administrative regions of Ghana, using a two-stage stratified cluster probability sampling design. The study used chi-square and modified Poisson with Generalized Estimating Equations (GEE) to examine help-seeking behavior among miscarrying women in Ghana and its impact on post-miscarriage health. The PMHP prevalence was 13.5% (95% CI: 12.0–15.1). Of the 1,843 women, 76.2% (95% CI: 74.3–78.2) sought help following a miscarriage, with 73.6% receiving help from healthcare professionals, 4.6% from non-healthcare professionals, and 21.8% receiving help from both groups. Help-seeking behavior was associated with factors such as education, place of residence, marital status, distance to a health facility, and money for treatment. Women who sought help had a 3.0% (Adjusted Prevalence Ratio, (aPR = 0.97, 95% Cl: 0.95–0.99) reduced prevalence of PMHP compared to those who did not seek help after controlling for other factors. Encouraging more women to seek help following a miscarriage could play a critical role in reducing PMHP, which can substantially improve their physical well-being. This finding highlights the need for more health education programs that address potential barriers in women at higher risk of miscarriage-related complications, including those aged ≥ 31 years, from seeking help after a miscarriage.

## Introduction

Miscarriage refers to the loss of a pregnancy before the fetus is viable [1] and is a common occurrence in 10–15% of all clinical pregnancies [2]. Most miscarriages, around 80%, occur during the first trimester of pregnancy [3]. The physical and psychological burdens of miscarriage can impact the couple, family, and attending physicians [4, 5]. While most miscarriages are unavoidable, particularly in genetically susceptible women [6], seeking help such as counseling during or after pregnancy has decreased or ameliorated physical and mental health issues [7]. However, in resource-limited countries, such as sub-Saharan Africa, stigma, shame, guilt, and superstition can prevent women from seeking help from appropriate institutions [8]. Such barriers could promote self-management of the devastating experiences of miscarriage from being publicly discussed. Instrumental support, such as financial assistance and healthcare accessibility, and sociodemographic factors, such as education and age, can influence help-seeking behaviors and impact post-miscarriage health problems (PMHP).

In Ghana, government health facilities and private organizations, including “MARIE STOPES Ghana,” offer counseling services aimed at reducing PMHP risks [9]. Miscarrying women in Ghana are often provided with various forms of social and psychological support from family, friends, and peer visitors who have personal experience with miscarriage. For many resource-limited countries, such as Ghana, achieving the Sustainable Development Goal (SDG)-3 [10] involves recognizing the essential role of enhancing maternal health and well-being.

However, there is no evidence of how help-seeking behavior following miscarriage has influenced PMHP in Ghana. The predominant focus of reports on PMHP [11, 12] is often centered on the causes and effects of this condition, with limited attention given to the influence of help-seeking behavior on PMHP. Understanding the impact of help-seeking behavior could provide valuable insights into designing effective management and prevention strategies. These strategies may include encouraging women to seek help after experiencing a miscarriage to prevent avoidable recurrent miscarriages and providing psychological and emotional support to help women cope with such traumatic experiences.

Therefore, the study sought to examine help-seeking behavior among miscarrying women in Ghana, whether it was sought from healthcare professionals or not, and its influence on PMHP. This information could help design effective management and preventive strategies to encourage affected women to seek help and emotionally support them following a miscarriage.

## Materials and methods

### Data source and study participants

**Fig 1:**
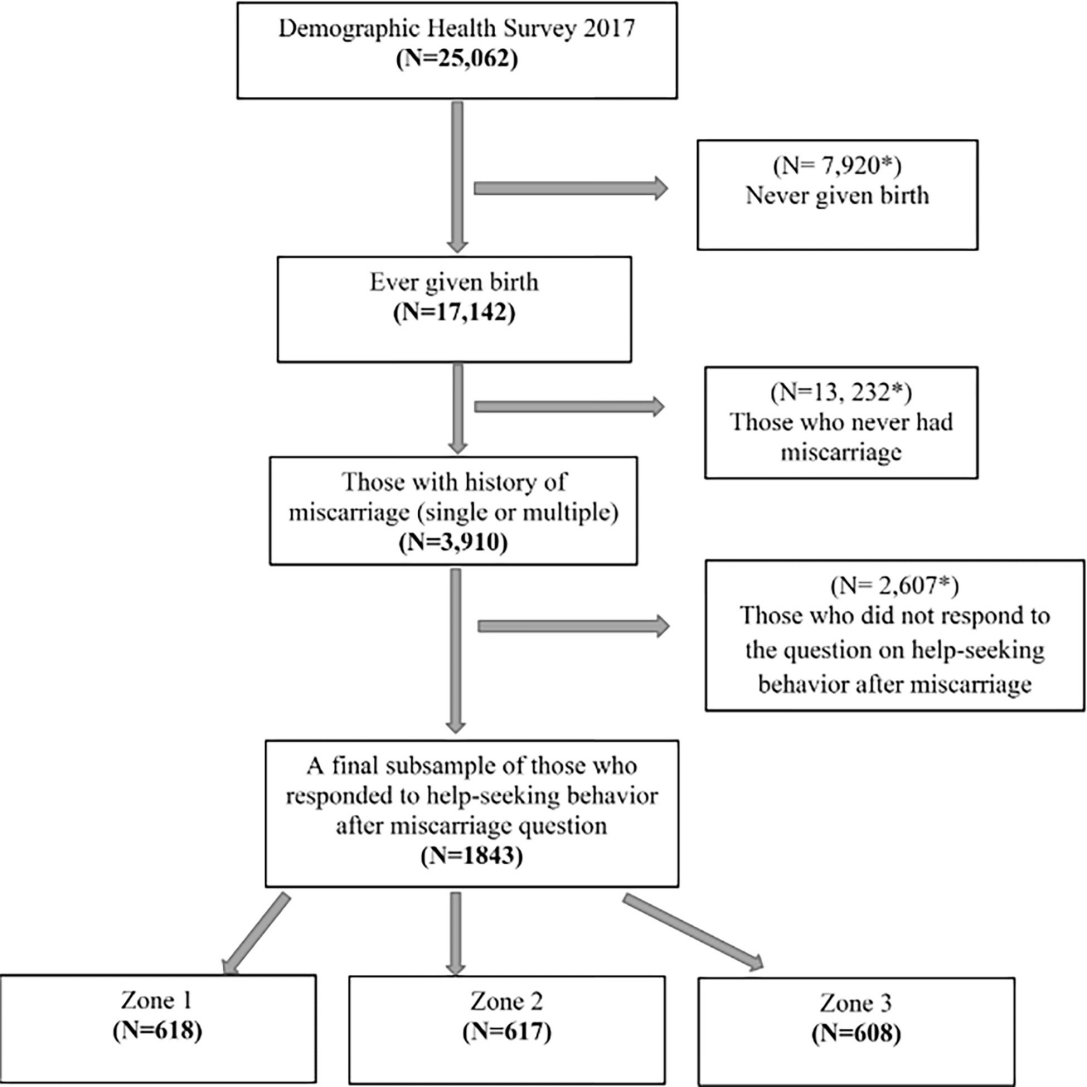
Restriction criteria for obtaining a sub-sample of DHS 2017 in this study.

The present study was based on data from the Ghana Maternal Health Survey [13]. This nationally representative cross-sectional survey was conducted between June 15th and October 12th, 2017, and provides information on maternal issues of women of reproductive age (15–49 years) within ten administrative regions and three zones in Ghana (Zone 1: Northern, Upper West, and Upper East; Zone 2: Ashanti, Eastern, and Brong Ahafo; Zone 3: Central, Greater Accra, Western, and Volta) [13]. An original sample of 25,062 participants was part of this survey. Out of this number, 3,910 respondents reported a history of miscarriage (both single and multiple). In this study, a final subsample of 1,843 women who responded to questions on help-seeking behavior after a miscarriage from 900 clusters via a two-stage stratified cluster probability sampling design was analyzed. The questionnaire used in the original survey was approved by the ICF Institutional Review Board, thereby ensuring ethics approval for data usage under the publicly available data clause, which exempts it from needing approval by a research ethics board [13]. Fig 1 below provides a detailed description of the criteria used to obtain the subsample of the DHS 2017 among miscarrying women of reproductive age in Ghana (samples excluded from the analysis are flagged asterisk).

## Measurements

### Primary outcome variable

The primary outcome variable for the present study was assessed based on the question, “In the first month after the miscarriage, did you have any health problems because of the miscarriage?” [13]. The health problems respondents were asked about (i.e., answer yes or no) included bleeding, pain, fever, injury/perforation, foul-smelling discharge, and others [13]. Bleeding, foul-smelling discharge, injury/perforation, and others, such as psychological stress are among the health complications and infections that have been linked to miscarriage in the literature [14, 15].

### Primary exposure variable

The exposure was help sought-following miscarriage and was measured as a binary indicator. This exposure variable was defined as any form of help/support received after miscarriage including social support (e.g., confiding in friends and family) [16], instrumental support (e.g., health services), and information support (e.g., information related to other support available to the miscarrying women) [17].

### Other predictors and covariates

There is evidence that several factors may directly or indirectly influence help-seeking behaviors [17–22]. These factors include age, education, place of residence [17–19, 21], distance to a health facility, and financial problems [22]. Hence, these factors were accounted for in the present study. Age was categorized into three groups (≤23 years, 24–30 years, and ≥31 years). Education attainment was categorized into two groups (No /Yes), with ‘No’ representing no formal education and ‘Yes’ referring to at least some level of formal education (e.g., primary, secondary, or tertiary education).

We also incorporated place of residence (rural/urban), marital status (currently married, cohabitating, not in a union), persons from whom help was sought after miscarriage (healthcare professionals, e.g., doctors, nurses, pharmacist /Non- healthcare professions, e.g., traditional birth attendant, traditional practitioner, and relative/friends) and care factors (Distance, no nearby health facility, not wanting to go alone, getting the money needed for treatment, getting permission to visit the doctor), all measured as a big problem/not a big problem into the present study. A ‘big problem’ represents significant difficulty in accessing the listed care factors, and ‘not a big problem’ refers to no significant barriers in accessing these care factors.

### Statistical analysis

The primary outcome and explanatory variables proportions were computed and reported to enhance understanding of the study demographics. A chi-square test [23] was performed to assess the association between help-seeking behavior and other explanatory variables. A modified Poisson regression was performed to examine the association between help-seeking behaviors and post-miscarriage health problems [24]. This procedure is helpful for situations where the outcome of interest is highly prevalent [25] and avoids overestimating the magnitude of the association.

The clustering effect from the survey sampling technique was accounted for using generalized estimating equations (GEEs) [26]. An exchangeable working correlation was assumed based on the smallest quasi-information criteria [27]. Hosmer’s (2000) model-building strategy was adopted for the study analyses [28]. All variables that satisfied the criteria of *p*<0.25 in the unadjusted analysis were included in the multivariate analysis [28]. A manual-backward elimination procedure was employed to identify and select the most contributing (*p*<0.05) explanatory variables for the study’s final adjusted model. A variable was considered a confounder if the adjusted estimate differed from the crude estimate by at least 20% [29].

## Results

**Fig 2:**
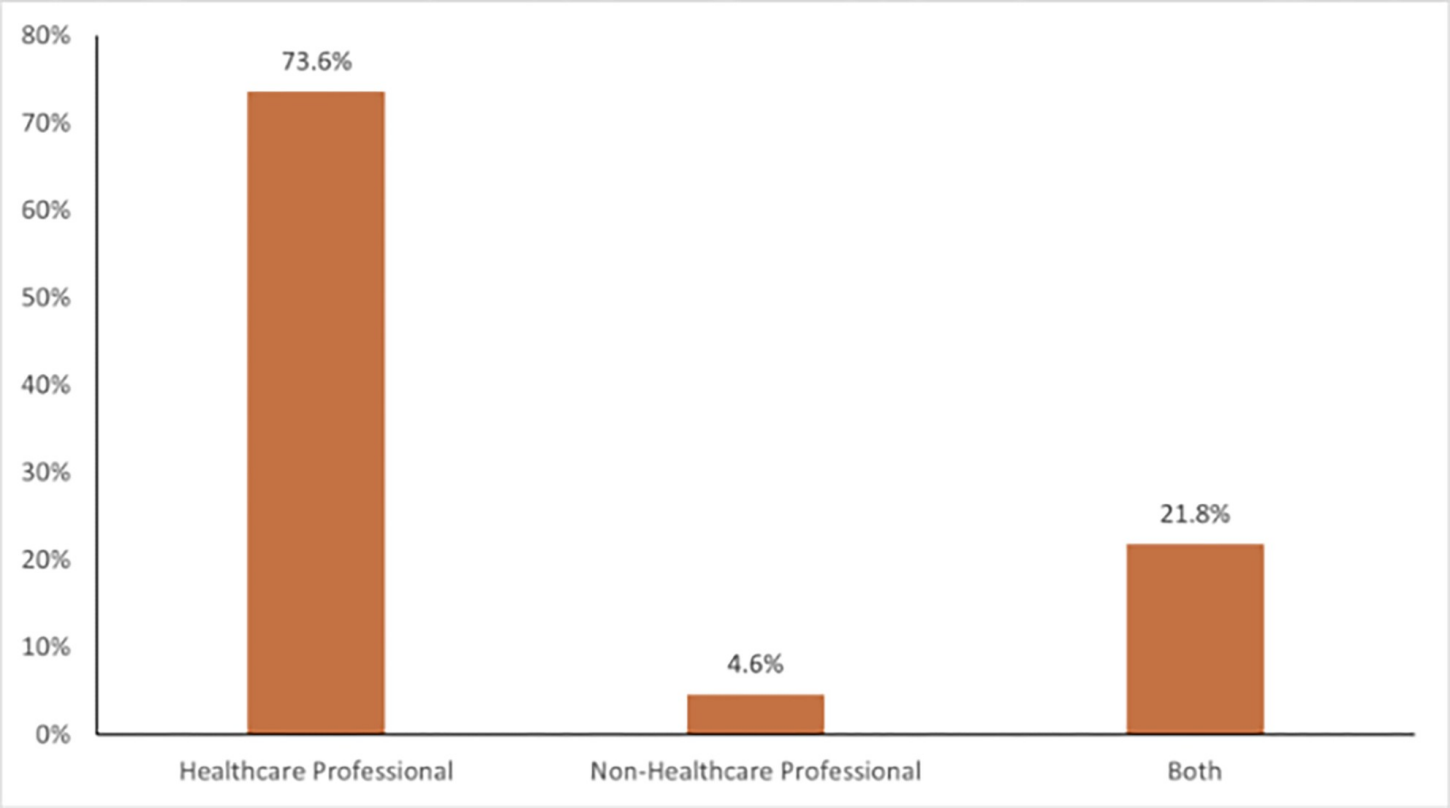
Persons who were approached for help after a miscarriage.

Our results show that, out of the 1843 women who responded to questions on miscarriage, approximately 13.5% (95% CI: 12.0–15.1) reported having health problems, with a majority, 76.2% (95% CI: 74.3–78.2) of the 1843 women, reported having sought help after miscarriage. Most (56.6%) of the miscarrying women who sought help had a single miscarriage (Table 1). Among those who sought help, the majority (73.6%) received help from healthcare professionals (e.g., doctors, nurses, pharmacists), whereas 4.6% went to non-healthcare professionals (e.g., traditional health attendants). In addition, 21.8% received a combination of help from both professionals (Fig 2). Also, women aged 31+ (39.4%) comprised the highest share of those seeking help, and 58.9% had received formal education. Those currently married formed the largest (47.6%) participant group surveyed, and approximately 40.9% resided in urban areas.

**Table 1:** Distribution of study participants demographics by help sought following a miscarriage (N = 1843).

Sought Help N (%)			
Variables	No	Yes	P-value*
**Age/years**			0.118
≤ 23	68 (3.7%)	174(9.5%)	
24–30	139(7.5%)	505(27.4%)	
≥ 31	231(12.5%)	726(39.4%)	
**Education**			0.002
No	132 (7.2%)	319 (17.3%)	
Yes	306 (16.6%)	1086 (58.9%)	
**Place of residence**			0.036
Rural	228(12.4%)	651(35.3%)	
Urban	210(11.4%)	754(40.9%)	
**Marital status**			<0.001
Currently married	216(11.7%)	877(47.6%)	
Cohabitating	144(7.8%)	321(17.4%)	
Not in union	78(4.2%)	207(11.3%)	
**Distance to a health facility**			0.022
Big Problem	130(7.1%)	340(18.4%)	
Not a Big Problem	308(16.7%)	1065(57.8%)	
**Not wanting to visit health facility alone**			0.171
Big Problem	72(3.9%)	194(10.5%)	
Not a Big Problem	366(19.9%)	1211(65.7%)	
**Getting money needed for treatment**			<0.001
Big Problem	248(13.5%)	648(35.1%)	
Not a Big Problem	190(10.3%)	757(41.1%)	
**Getting access to the doctor**			0.062
Big Problem	38(2.1%)	86(4.7%)	
Not a Big Problem	400(21.7%)	1319(71.5%)	
**Miscarriage episode**			0.054
Single	305 (16.6%)	1044 (56.6%)	
Multiple	133 (7.2%)	361(19.6%)	
**Health Problems**			0.011
Yes	43 (2.4%)	205 (11.1%)	
No	395 (21.4%)	1200 (65.1%)	

*P-value from the Chi-Square test.

About 58.0% of the participants who sought help reported that proximity to a health facility was not a major problem. Likewise, 65.7% did not have a significant problem getting someone to escort them to a health facility. More than two-thirds of those who sought help indicated that seeing a doctor was not a big problem (71.5%), and 41.1% reported getting money for treatment was a significant challenge for them. Education, place of residence, marital status, distance to a health facility, and money for treatment were associated with help-seeking behavior. In contrast, age, getting access to the doctor, and not wanting to visit a health facility alone were not associated with help-seeking behavior (Table 1).

### Demographic characteristics of study participants by post-miscarriage health problems

The distribution of demographic characteristics of study participants by post-miscarriage health problems is presented in Table 2. The results show that higher proportions of post-miscarriage health problems were observed in women with a history of single miscarriage (10.1%) compared to women with multiple episodes of miscarriage (3.4%). Also, the proportion of health problems following miscarriage increased as age increased (age ≤ 23 years: 1.6%; age 24–30 years: 4.7%; age ≥ 31 years: 7.2%). Women who had received formal education reported a higher proportion (10.8%) of post-miscarriage health problems. Compared to women in urban settings, women who resided in rural settings had a higher proportion of post-marriage health problems (rural 7.3% vs urban 6.2%). The proportions of post-miscarriage health problems were higher among married women (8.1%) and women who were financially constrained (7.1%). Compared to their counterparts, women who did not consider distance to a health facility, access to physicians, and the need for accompaniment to a health facility as significant problems reported a higher proportion of post-miscarriage health issues.

**Table 2:** Distribution of demographic characteristics by post-miscarriage health problems following a miscarriage (N = 1843).

Variables	Health Problem	
	No	Yes
**Miscarriage episode**		
Single	1164 (63.2%)	185 (10.1%)
Multiple	431 (23.3%)	63 (3.4%)
**Age/years**		
≤ 23	213 (11.6%)	29 (1.6%)
24–30	558 (30.3%)	86 (4.7%)
≥ 31	824 (44.7%)	133 (7.2%)
**Education**		
No	402 (21.8%)	49 (2.7%)
Yes	1193 (64.7%)	199 (10.8%)
**Place of residence**		
Rural	744 (40.4%)	135 (7.3%)
Urban	851 (46.1%)	113 (6.2%)
**Marital status**		
Currently married	945 (51.2%)	148 (8.1%)
Cohabitating	404 (21.9%)	61 (3.3%)
Not in union	246 (13.3%)	39 (2.1%)
**Distance to a health facility**		
Big Problem	403 (21.8%)	67 (3.7%)
Not a Big Problem	1192 (64.7%)	181 (9.8%)
**Not wanting to visit health facilities alone**		
Big Problem	228 (12.3%)	38 (2.1%)
Not a Big Problem	1367 (74.2%)	210 (11.4%)
**Getting money needed for treatment**		
Big Problem	765 (41.5%)	131 (7.1%)
Not a Big Problem	830 (45.0%)	117 (6.4%)
**Getting access to the doctor**		
Big Problem	103 (5.6%)	21 (1.2%)
Not a Big Problem	1492 (80.9%)	227 (12.3%)

### Unadjusted and adjusted associations between predictor variables and outcome

Table 3 summarizes the results of both unadjusted and adjusted analyses. A statistically significant association was found between help-seeking and post-miscarriage health problems. After adjusting for potential confounding variables, women who sought help had their prevalence of post-miscarriage health problems reduced by 3.0% (aPR = 0.97, 95% Cl 0.96–0.99) compared to those who did not seek help. In addition, the results revealed that education is significantly associated with post-miscarriage health problems, with a 2.0% (aPR = 0.98, 95% Cl 0.96–0.99) lower prevalence observed in those who had formal education than those who did not.

**Table 3:** Unadjusted and adjusted associations of post-miscarriage health problems, help-seeking and other factors.

	Unadjusted Model		Adjusted Model
Variables	uPR [95%Cl]	Overall P-values	aPR [95%Cl]
**Sought Help for Miscarriage**			
No (ref)	1.00		
Yes	0.97[0.96–0.99]	0.005	0.97[0.95–0.99]
**Age/years**			
≤ 23 (ref)	1.00		1.00
24-30	1.00[0.97–1.02]		1.00[0.98–1.02]
≥ 31	1.01[0.98–1.03]	0.241	1.03[1.01–1.06]
**Education**			
No (ref)	1.00		1.00
Yes	0.98[0.96–0.99]	0.045	0.97[0.95–0.99]
**Place of residence**			
Rural (ref)	1.00		1.00
Urban	1.02[1.01–1.04]	0.023	1.03[1.01–1.05]
**Marital status**			
Not in union (ref)	1.00		
Currently married	1.00[0.98–1.02]		
Cohabitating	0.99[0.96–1.02]	0.842	
**Distance to a health facility**			
Not a Big Problem (ref)	1.00		
Big Problem	0.99[0.97–1.01]	0.569	
**Not wanting to visit health facility alone**			
Not a Big Problem (ref)	1.00		
Big Problem	1.00[0.96–1.02]	0.687	
**Getting money needed for treatment**			
Not a Big Problem (ref)	1.00		
Big Problem	0.98[0.97–1.00]	0.141	
**Getting access to the doctor**			
Not a Big Problem (ref)	1.00		
Big Problem	0.98[0.95-1.02]	0.273	

uPR: Unadjusted Prevalence Ratio; aPR: Adjusted Prevalence Ratio; Cl: Confidence Interval

The results also show that the prevalence of post-miscarriage health problems was 1.03 times greater among women in urban than those in rural Ghana. Moreover, no significant difference in the prevalence of post-miscarriage health problems was identified between those aged 15–23 years and 24–30 years (aPR = 1.00, 95% Cl 0.98–1.02). However, an increased prevalence of post-miscarriage health problems was observed in women aged 31+ years. Thus, women aged 31+ years were at a 1.03 times greater prevalence of post-miscarriage health problems than those aged 15–23 years (aPR = 1.03, 95% CI: 1.01–1.06).

## Discussion

According to the findings of the study, seeking support services after a miscarriage was a significant way to minimize post-miscarriage health issues. In this study population, a lower occurrence of post-miscarriage health problems was associated with having a formal education. The study also revealed that women in rural areas were less likely to experience post-miscarriage health problems than those in urban areas. Compared to women aged 15–23 years, post-miscarriage health problems were significantly higher in those aged 31 years or older. There was no significant variation between the age groups of 15–23 and 24–30 years. The results from the chi-square analysis indicated that seeking help was associated with education, place of residence, marital status, distance to a health facility, and access to funds for treatment.

Previous research found that seeking both formal and informal help after a miscarriage reduces post-miscarriage health issues [30, 31]. Studies by Séjourné et al. and Alqassim et al. reported significant improvement in health outcomes among women who received various forms of support and help following a miscarriage, including peer counseling, informal social support, and medical consultation [30, 31]. The results of the present study were consistent with these findings, as seeking help after a miscarriage was associated with a 3.0% decrease in the prevalence of post-miscarriage health problems.

Furthermore, education was found to be a factor that encouraged women who experienced a miscarriage to seek help and subsequently lower their risk of post-miscarriage health issues [32, 33]. The current study also revealed that women with higher formal education were more likely to seek help, and their post-miscarriage health problem prevalence was significantly reduced by 2.0% compared to those without formal education. This aligns with the findings of a recent systematic review, which examined post-miscarriage care, and supports the notion that individuals with a higher level of education who experience a spontaneous miscarriage are more inclined to seek assistance compared to those with lower levels of education [34]. Additionally, a study by Jensen et al. showed that women who received meditation and mindfulness training following a miscarriage experienced a significant reduction in stress levels [35]. These findings suggest that miscarriage-related education is essential in preventing future post-miscarriage complications and avoiding recurrent miscarriages.

The availability of support and care following a miscarriage could influence a woman’s ability to reduce the risk of post-miscarriage health problems [36]. However, access to obstetric and gynecological services may be limited in remote areas of resource-limited countries [37]. The study revealed that urban women were more likely to experience post-miscarriage health problems than women in rural areas, but misclassification of place of residence is possible. The social support system, including extended family and friends, is more functional and effective in rural areas, which may account for the relatively lower prevalence of post-miscarriage health problems in rural dwellers.

On the other hand, the lower prevalence of post-miscarriage health problems in women residing in rural areas may result from underreporting the incidence of post-miscarriage health problems compared to their counterparts in the urban setting. Dellicour et al. [36] found that women in rural areas infrequently seek support/care after miscarriage unless problems set in [36]. A more rigorous study design, including prospective cohort studies, would be needed to clarify the association between place of residence and post-miscarriage health problems. Women older than 31 years were found to be more likely to experience post-miscarriage health problems, which is not surprising given their higher risk of miscarriage [38, 39].

Although factors such as marital status, distance to a health facility, and money for treatment were not significantly associated with post-miscarriage health problems in the study subjects, they significantly influenced health-seeking behaviors [22, 40]. Financial problems and distance to a health facility have been found to be associated with help-seeking behavior in previous studies [22]. The emotional support and physical presence of a woman’s partner following a miscarriage positively influence their recovery [40].

### Strengths and limitations

We employed a robust statistical method that considered the complexity of the sampling procedure and used a nationally representative sample to assess the impact of help-seeking on post-miscarriage health problems. However, despite these notable strengths, the study has some limitations. First and foremost, we were unable to determine the independent effect of informational, social, and instrumental support/help on post-miscarriage health problems. Furthermore, we could not assess the influence of factors such as depression, anxiety, and treatment costs on post-miscarriage health problems, as we lacked the necessary data. Additionally, the self-reported miscarriage without confirmation of pregnancy status through a urine pregnancy test could be misleading, as some women may consider a delayed start of their menses a miscarriage. Due to limited data on women with multiple miscarriages, this study could not stratify the analysis by miscarriage episodes to further examine the differential impact of help-seeking behavior on post-miscarriage health problems among single and multiple miscarrying women. Also, the fact that only 47% of all women who experienced a miscarriage responded to these questions is a potential source of selection bias. Further, due to the composite nature of the exposure variable, it was not possible to separately assess the influence of social support in comparison to health service support on help-seeking and the risk of post-miscarriage health problems. Finally, the composite nature of the outcome variable also made it challenging to quantify disparities in the severity of different outcomes, such as foul-smelling discharge, injury, or perforation resulting from a miscarriage.

## Conclusion

Promoting the utilization of support services among women following a miscarriage can contribute to lessening the impact of post-miscarriage health issues. Implementing health education initiatives aimed at addressing potential obstacles faced by women, particularly those aged 31 years and above, in seeking assistance after a miscarriage, can significantly enhance their overall physical and emotional health and well-being. These broad findings linking the impact of help-seeking and the risk of post-miscarriage health problems provide an opportunity for subsequent waves of the DHS survey to incorporate important variables; help sought through social support and health service support. This would help inform which support system clinically/substantively drives the association.

## Supporting information

S1 Checklist STROBE statement—a checklist of items that should be included in reports of observational studies. (DOCX)
